# Novel insights into the classification of Shamblin III carotid body tumors from a neurosurgical perspective

**DOI:** 10.1007/s10143-024-02389-x

**Published:** 2024-04-05

**Authors:** Qianquan Ma, Yu Si, Mingyang Sun, Wanzhong Yuan, Chao Wu, Yunfeng Han, Xiaoliang Yin, Jun Yang, Tao Wang

**Affiliations:** 1 Department of Neurosurgery, Peking University Third Hospital, Peking University, Beijing, 100191 China; 2 Department of Neurosurgery, Tangshan People’s hospital, Tangshan, Hebei 063001 China

**Keywords:** Carotid body tumor, Shamblin classification, Parapharyngeal space, Arterial-relevant classification, Anatomical-relevant classification

## Abstract

**Background and purpose:**

The classic Shamblin system fails to provide valuable guidance in many Shamblin’s III carotid body tumors (III-CBTs) due to the variable forms of carotid arteries and the complex anatomic relationships in parapharyngeal space. We proposed a modified classification to separately divide III-CBTs into different subgroups on the basis of arterial relevant features and anatomical relevant features.

**Materials and methods:**

From 2020 to 2023, a total of 129 III-CBTs at a single institution were retrospectively analyzed. All cases were independently classified as arterial-relevant and anatomical-relevant subgroups. The pre-, peri- and postoperative data were summarized and compared accordingly.

**Results:**

Among the 129 cases, 69 cases were identified as “Classical type”, 23 cases as “Medial type”, 27 cases as “Lateral type” and 10 cases as “Enveloped type” according to arterial morphologies. Besides, 76 cases were identified as “Common type”, 15 cases as “Pharynx- invasion type”, 18 cases as “Skull base-invasion type” and 20 cases as “Mixed type” according to anatomical relationships. “Enveloped type” of tumors in arterial-relevant classification and “Mixed type” of tumors in anatomical-relevant classification are the most challenging cases for surgeons with the lowest resection rate, highest incidence of carotid arteries injury and postoperative stroke.

**Conclusion:**

The modified classifications provide comprehensive understanding of different III-CBTs which are applicable for individualized treatment in clinical practice.

## Introduction

Carotid body tumors (CBTs), also known as carotid body paragangliomas, are rare, benign, and highly vascular tumors with an incidence of less than 1:30000 in the general population [1, 2]. CBTs arise from chromaffin cells and are associated with the autonomic paraganglia. These tumors are often biochemically silent, but sometimes display neuroendocrine secretions of histamine, serotonin, epinephrine and norepinephrine [3]. Originating from the carotid bifurcation, CBTs slowly develop in the parapharyngeal space (PPS) with encasement of the common carotid artery (CCA), external carotid artery (ECA), and internal carotid artery (ICA), and compression of the IX-XII cranial nerves. Due to the relatively large space of the PPS, lesions are always detected by coincidence or posterior cranial nerve dysfunction. A significant number of tumors can expand to a large extent without presentation, creating an extremely high risk for surgical treatment.

Because of their distinct relationship with the carotid arteries and localized expansion in the lateral skull base, CBTs are removed by vascular surgeons, ENT surgeons, and neurosurgeons with different specializations. Due to the severe adhesion of adjacent vessels and cranial nerves, surgery can be complicated by massive blood loss, lethal postoperative stroke, and neurological impairment [4]. Active strategies such as preoperative embolization, carotid artery stenting, intraoperative ICA reconstruction, chemotherapy, and radiotherapy have been introduced for the optimal treatment of CBTs [4–8]. However, with advancements in imaging technology and operative skills, surgical resection remains the preferred option for CBTs, with significantly reduced complications and mortalities [9].

The Shamblin classification was first used in the 1970s to assess the relationship between CBTs and carotid vessels and is globally applied in clinical practice [10]. Tumors with no, partial, or complete encasement of the carotid arteries are classified into subgroups I, II, and III [10]. However, the Shamblin system only focuses on vascular associations without considering detailed arterial morphology and distinguishing anatomical invasion within the PPS, especially in Shamblin group III CBTs (III-CBTs). Studies have attempted to modify the traditional Shamblin system to better understand the relationship between the distinctive shapes of the CBTs and different clinical outcomes [6, 11, 12]. One team of vascular surgeons provided a modified classification to illustrate the vertical correlation between the tumors and skull base structures [11]. Our team has one of the largest annual cohorts of CBT operations in China. We previously analyzed the risk factors for neurological symptoms after CBT resection in our patients [13]. In this study, we aimed to further clarify the specific arterial forms within tumors and demonstrate the varied anatomic differences between III-CBTs. This novel classification will enhance the understanding of tumors and provide valuable guidance for clinical practice.

## Patients and methods

### Patients

This retrospective study only enrolled patients with III-CBTs. From 2020 to 2023, patients with III-CBT underwent computed tomography angiography (CTA) and carotid high-resolution MRI (h-MRI) within one week before surgery. Patients with complicated tumors also underwent preoperative digital subtraction angiography (DSA) and a balloon occlusion test (BOT) to examine the function of the circle of Willis and intracranial blood perfusion. All patients underwent microsurgical treatment, as performed by Professor Tao Wang. CTA was performed 24 h after surgery to observe vessel patency. H-MRI was performed 48 h after surgery to determine the extent of resection. Patients with bilateral tumors underwent unilateral surgery for the smaller tumor first, and the opposite-side tumor was treated 3–6 months later. Bilateral tumors in a single patient were analyzed separately. No preoperative endovascular embolization or stent implantation was performed.

### Microsurgical procedure

**Fig. 1:**
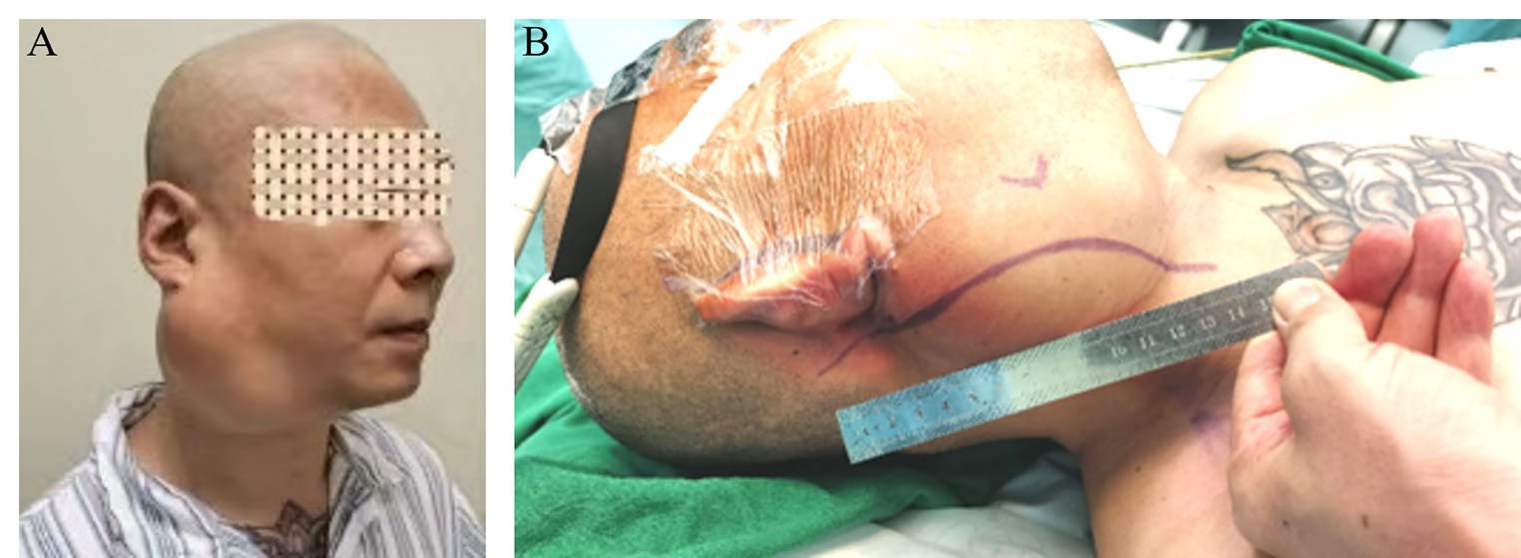
(**A**) Diagram of one patient with huge III-CBT which was classified as “Mixed type”. The tumor invaded both the skull base and pharynx as shown in picture. (**B**) The curved incision along the anterior border of SCM covered the upper and lower end of tumor. The postauricular end of incision was upper than mastoid process to completely expose the skull base

A regular transcervical approach with a curved incision along the anterior border of the sternocleidomastoid (SCM) muscle from the mandibular angle to the sternal angle was used in each case according to the tumor size (Fig. 1). Every operation started with exposure of the proximal end of the CCA. After exposing and marking the CCA, the surgeon further separated the medial and lateral boundaries of the tumor distally. The internal jugular vein was properly protected when the lateral boundary was separated. The upper end or skull base part of the tumor was carefully dissected under high magnification. If the tumor extended upward and invaded the skull base, complete removal of the tumor was not possible, and the surgical strategy was switched to piecemeal resection. In III-CBTs, both the ICA and ECA are surrounded by the tumor capsule or tumor tissue. The relative locations of the ICA and the ECA differed in each case. Separation of the ECA or ECA branches within the tumor was attempted at first, and the ECA was followed to the bifurcation of the CCA. The ICA was also separated from the distal to proximal end after the tumor was dissected from the ECA. Tissue of tumor along the bifurcation was often regarded as the most cohesive part and should be separated at last.

Comprehensive preoperative examinations are essential to assess the risk of surgery. MRI and ultrasound were effective in predicting tumor consistency. If the tumors were hard in texture and the carotid arteries (especially the ICA) were entirely encased within the tumor, great attention was given to avoid massive bleeding, and complete solutions were prepared if the ICA required revascularization. Various strategies were used to manage massive intraoperative arterial bleeding. Small-artery ruptures were repaired using 10 − 0 prolene vascular sutures. Uncontrollable ECA bleeding was treated by ECA ligation. Severe ICA bleeding should be avoided as much as possible. If this occurred, bleeding was controlled by ligation of the ICA with or without reconstruction of the ICA. Revascularization methods, such as vascular patches (AESCULAP, No.1,107,291) or artificial vessels (GORE-TEX), were used according to the intraoperative conditions.

### Peking University (PKU) classification

The updated PKU classification divides III-CBTs into two independent systems to aid surgeons in understanding tumor characteristics from different perspectives.

1. Classification based on the location of carotid arteries within the tumor (arterial-relevant classification);
2. Classification depending on the degree of tumor invasion of anatomic structure in the PPS (anatomical-relevant classification).

#### Arterial-relevant classification

**Fig. 2:**
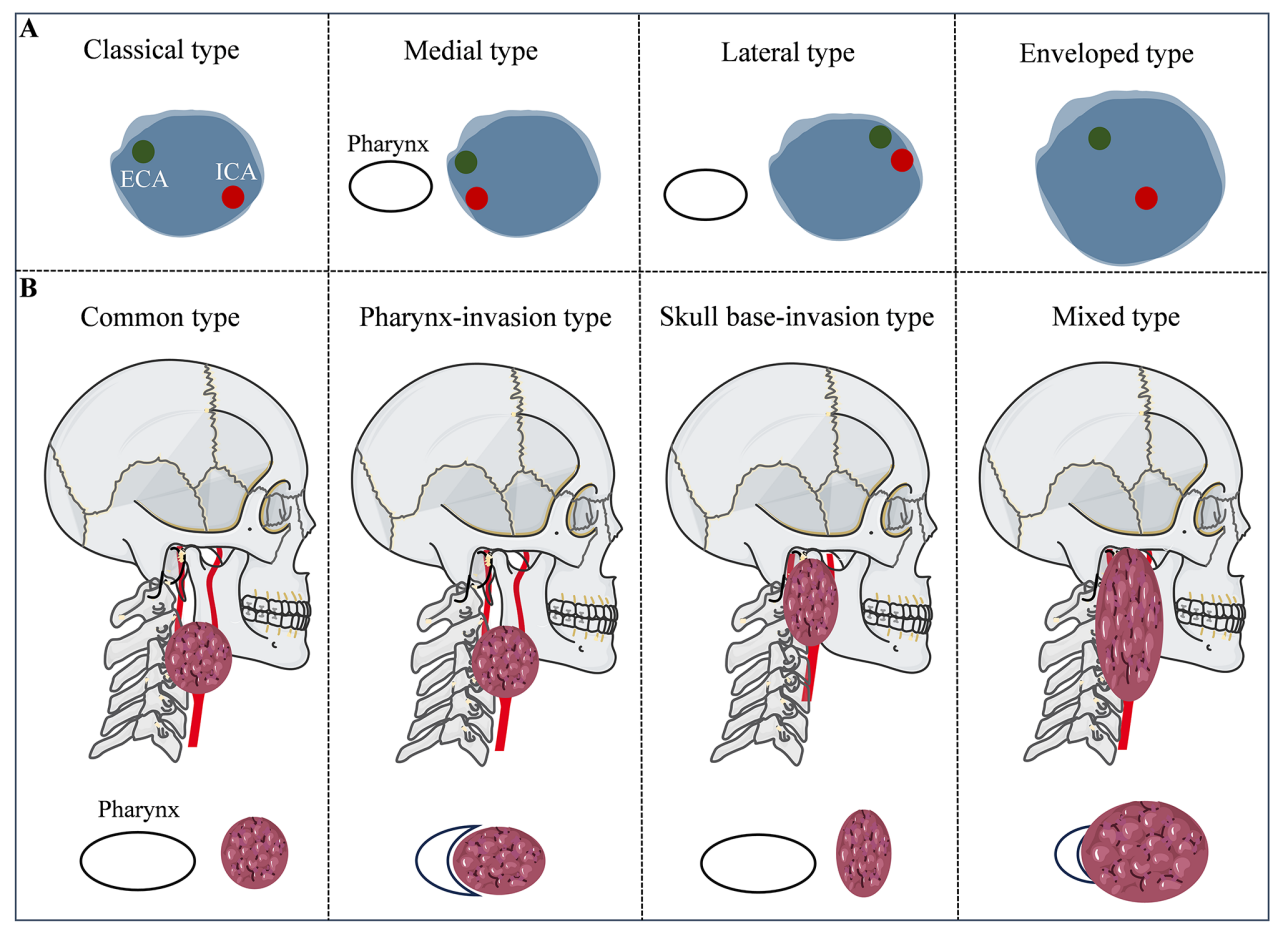
PKU classifications of III-CBTs. (**A**) Arterial-relevant classification. (**B**) Anatomical-relevant classification

**Fig. 3:**
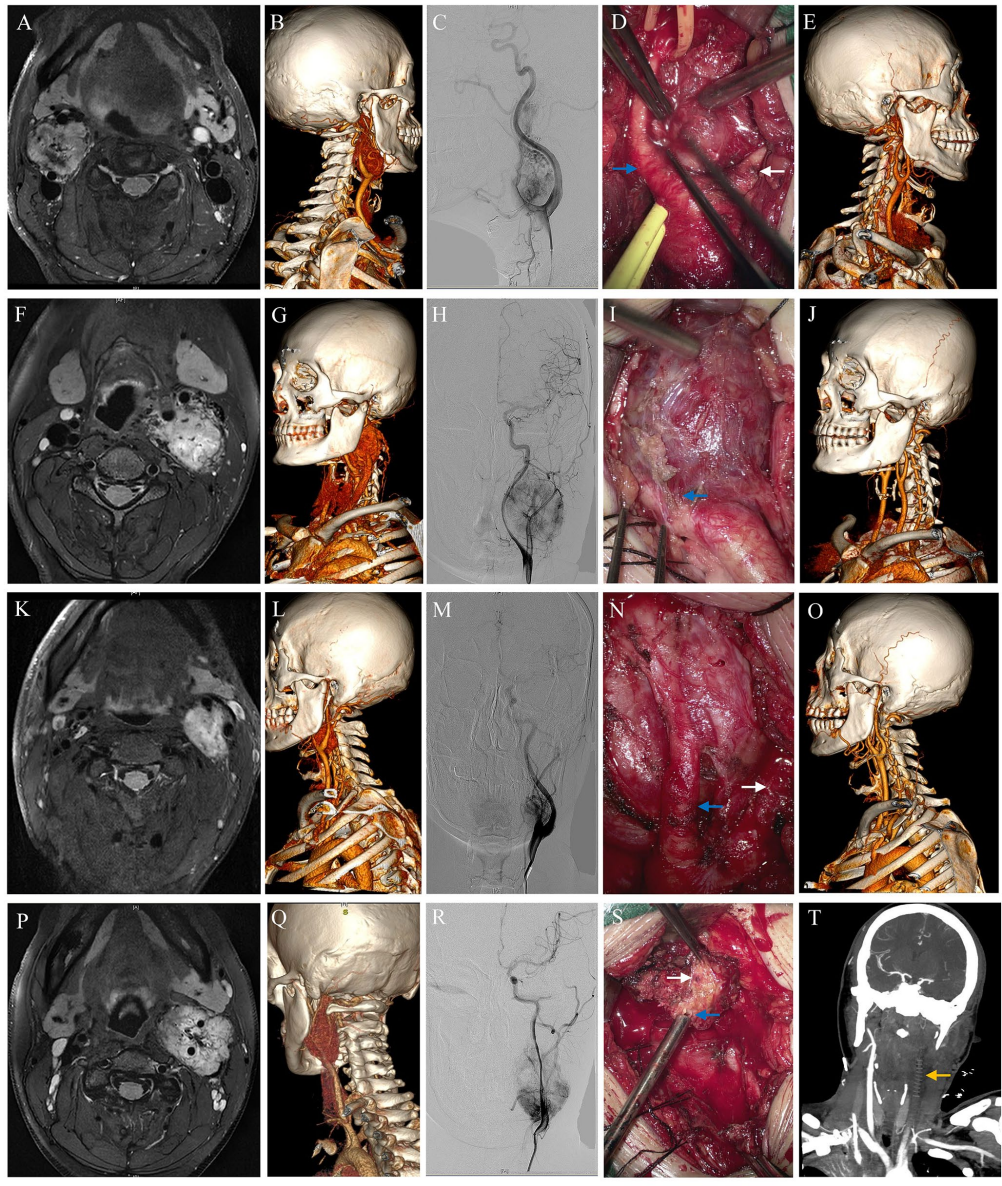
The preoperative MRI, CTA and DSA, intraoperative image and postoperative CTA images of different subtypes of tumors based on arterial relevant classification. **A-E**. Classical type. **F-J**. Medial type. **K-O**. Lateral type. **P-T**. Enveloped type. White arrow: ECA. Blue arrow: ICA. Yellow arrow: artificial vessel. The artificial vessel was used for ICA reconstruction but failed due to intravascular thrombosis

Based on the different morphologies of the carotid arteries within the tumors, III-CBTs are divided into four subgroups: classical, medial, lateral, and enveloped (Figs. 2A and 3).

##### Classical type

The CCA is located in the caudal portion of the tumor. The ECA and ICA are separated by the tumor bulk and are located on either side. The ECA is usually located anteromedially and the ICA is usually located posterolaterally to the tumor.

##### Medial type

The CCA is located in the caudal part of the tumor. The ECA and ICA are adjacent to each other and located medially to the tumor mass.

##### Lateral type

The CCA is located in the caudal part of the tumor. The ECA and ICA are adjacent to each other and are located lateral to the tumor mass.

##### Enveloped type

The ECA, ICA, or both, and the upper part of the CCA are completely encased within the tumor.

#### Anatomical-relevant classification

**Fig. 4:**
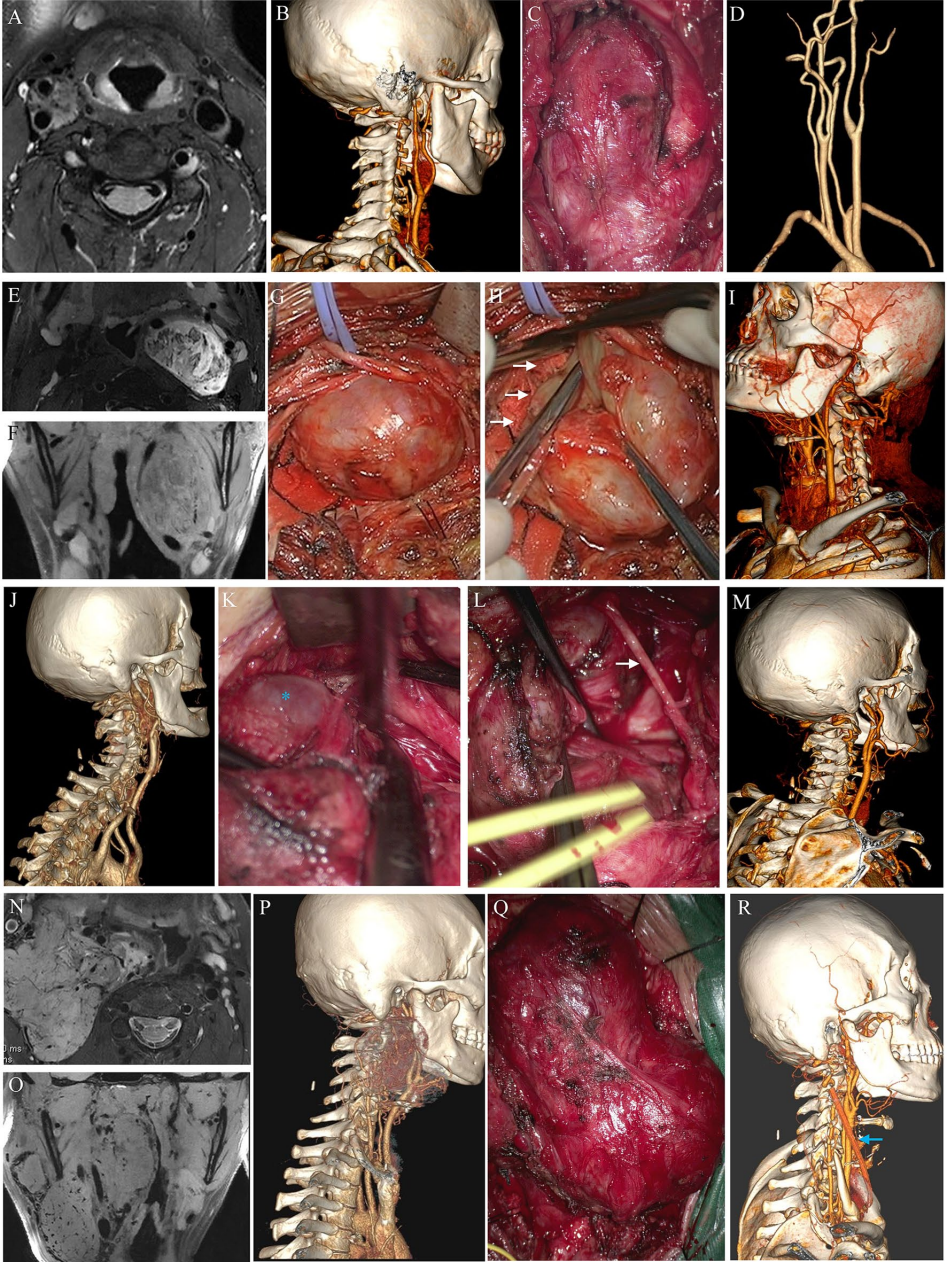
The preoperative MRI, CTA, intraoperative image and postoperative CTA images of different subtypes of tumors based on anatomical relevant classification. **A-D**. Common type. **E-I**. Pharynx-invasion type. **E, F**. The MRI axial and coronal view of tumor. The lateral wall of pharyngeal cavity was obviously suppressed by tumor. **G, H**. The main body of tumor was dragged out to expose the lateral wall of pharyngeal cavity (white arrow). Brain cotton slices were selected to protect pharynx wall. **J-M**. Skull base-invasion type. **K**. Tumor near the jugular foramen was carefully resected under high magnification. *: The jugular vein which just exits the skull base. **L**. The main body of tumor was dragged down to expose hypoglossal nerve (white arrow). **N-R**. A giant “Mixed type” of tumor invaded both the skull base and pharynx. Blue arrow: drainage tube

Based on the invasion of the tumor into adjacent anatomical structures in the PPS, III-CBTs are classified into four subgroups: common type, pharynx-invasion type, skull base-invasion type, and mixed type (Figs. 2B and 4).

##### Common type

The most regular type of III-CBT. The superior margin of the tumor is below the C2 level without invasion of the skull base structures, such as the mastoid process, styloid process, or jugular foramen. The pharyngeal cavity is unaffected by the tumor. The pharynx is morphologically normal and symmetrical based on MRI evaluation.

##### Pharynx-invasion type

The tumor is below the C2 level, but grows in a boot shape to invade the pharynx medially. The pharynx is then compressed and narrowed.

##### Skull base-invasion type

The tumor grows upward along the ICA and internal jugular vein to reach the skull base. The superior margin of the tumor is above the C1 level. Sometimes, the upper part of the tumor is higher than the lower edge of the mastoid or styloid process. The space between the internal jugular foramen, mastoid process, and styloid process is occupied by the tumor.

##### Mixed type

The mixed type is a combination of pharynx-invasion and skull base-invasion types. The tumor is usually large and exhibits significant invasiveness. The skull base and pharynx are simultaneously invaded.

#### Follow-up

All patients were followed up at the outpatient center for six months. The main postoperative complications included neurological dysfunction (hoarseness, loss of voice, choking, tongue extension deviation, and Horner syndrome), neck hematoma, wound infection, tumor recurrence, and cerebrovascular accidents. Long-term complications have been described in our previous study [13].This present study only considered the short-term complications immediately after surgery.

### Statistical analysis

Data analyses were performed using SPSS 27.0. The pre- and postoperative clinical data in the different subgroups were compared using one-way analysis of variance. Data were expressed as mean ± SD. Statistical significance was set at *p* < 0.05.

#### Ethics committee approval

This study was approved by the ethics committee of our hospital and conducted according to the principles of the Declaration of Helsinki.

## Results

This study enrolled 129 patients who underwent treatment for III-CBTs. The tumors were subdivided into subgroups based on the PKU classification system. The bilateral tumors in a single patient were analyzed separately.

### Clinical information (arterial-relevant classification)

According to the arterial-relevant classification, 69 cases were identified as classical type, 23 cases as medial type, 27 cases as lateral type and 10 cases as enveloped type. No significant differences were found in age, sex, site, or preclinical symptoms among the four groups. A total of 117 patients received first-time treatment at our hospital. The remaining 12 patients were first treated in other hospitals and then underwent a second or third operation in our hospital. The most common preclinical symptoms were asymptomatic masses, followed by posterior cranial nerve dysfunction, and tongue deviation (Table 1).

**Table 1:** Clinical characters of arterial-relevant classification

Total:129 cases	Classical Type (*n* = 69)	Medial Type (*n* = 23)	Lateral Type (*n* = 27)	Enveloped Type (*n* = 10)	*P*-value
Age, years	44.71 ± 12.05	44.04 ± 13.39	45.41 ± 12.90	37.40 ± 13.79	0.355
Female/Male	35/34	16/7	14/13	5/5	0.448
Site (left/right)	37/32	10/13	17/10	6/4	0.346
Initial surgery	62 (89.86)	21 (91.30)	25 (92.59)	9 (90.00)	
Non-initial surgery	7 (10.14)	2 (8.70)	2 (7.41)	1 (10.00)	
Asymptomatic mass	28 (40.58)	11 (47.83)	15 (55.56)	4 (40.00)	0.586
Throat pain or discomfort	23 (33.33)	6 (26.09)	5 (18.52)	4 (40.00)	0.438
IX-XI cranial nerves dysfunction (Hoarseness, dysphagia, cough when drinking)	29 (42.03)	8 (34.78)	8 (29.63)	2 (20.00)	0.446
Tongue deviation	4 (5.80)	1 (4.34)	1 (3.70)	1 (10.00)	0.889

### Surgical outcomes (arterial-relevant classification)

The same transcervical approach was used for each patient, and the incision length differed according to tumor size. Representative pre-, intra-, and postoperative images of the different subgroups are shown in Fig. 3. The microsurgical results based on the arterial classification are summarized in Table 2. Most tumors were completely resected, and only 16 were partially resected. In subtotal resection cases, most of the tumor (> 95%) was removed, and the residual tumor at the bifurcation or skull base site was inactivated by electrocoagulation. The total resection rates in the four subgroups were similar. However, the average bleeding, operation time, length of hospitalization, intensive care unit (ICU) care, stroke incidence and ECA/ICA ligation rates were significantly higher in enveloped group. Because the initial step was to attempt separation of the tumor from the ECA or ECA branches, and the operative space was narrow and cramped, ECA rupture and ligation were relatively frequent. A total 19 ECAs were ligated, with six cases in enveloped group. The enveloped group also included all the patients in which ICA ligation was required. A vascular patch was applied in six cases when the ICA was partially torn. In five cases requiring ICA ligation, three received reconstruction using artificial vessels. One patient experienced acute ICA occlusion and underwent endovascular stenting. All five patients with ICA ligation underwent postoperative ICU care to avoid cerebrovascular accidents. Six patients in the other groups also underwent ICU care because of their relatively old age, difficult airway management, and other comorbidities. Postoperative cerebral infarctions occurred in three patients in enveloped group. One case of cerebral infarction was due to ligation of the ICA, and the other two cases were due to thrombogenesis in the artificial vessels. Newly developed neurological dysfunctions immediately after surgery are listed in Table 2. Secondary lung infections were relatively frequent because of posterior cranial nerve impairment. No severe hematoma or cardiovascular accidents were detected in our cohort.

**Table 2:** Surgical outcomes based on arterial-relevant classification

Total:129 cases	Classical Type (*n* = 69)	Medial Type (*n* = 23)	Lateral Type (*n* = 27)	Enveloped Type (*n* = 10)	*P*-value
Total resection	60 (86.96)	21 (91.30)	25 (95.59)	7 (70.00)	0.285
Subtotal resection	9 (13.04)	2 (8.70)	2 (7.41)	3 (30.00)	0.285
ECA ligation	8 (11.59)	4 (17.39)	1 (3.70)	6 (60.00)	<0.001
ICA ligation	0	0	0	5 (50.00)	<0.001
ICA Vascular patch	1 (1.45)	2 (8.70)	0	3 (30.00)	<0.001
ICA reconstruction (Artificial vessel)	0	0	0	3 (30.00)	<0.001
ICA reconstruction (Interventional stent)	0	0	0	1 (10.00)	0.007
Bleeding, ml	56.55 ± 95.91	193.48 ± 215.57	65.37 ± 64.67	350.00 ± 141.42	<0.001
Operation time, min	178.70 ± 91.80	299.57 ± 191.61	194.70 ± 87.95	446.20 ± 157.96	<0.001
ICU care	3 (4.35)	2 (8.70)	1 (3.70)	5 (50.00)	<0.001
Stroke	0	0	0	3 (30.00)	<0.001
IX-XI cranial nerves dysfunction (Hoarseness, dysphagia, cough when drinking)	13 (18.84)	4 (17.39)	3 (11.11)	4 (40.00)	0.256
Tongue deviation	4 (5.80)	1 (4.35)	1 (3.70)	2 (20.00)	0.001
Lung infection	6 (8.70)	0	2 (7.41)	2 (20.00)	0.249
Length of hospitalization, day	11.13 ± 5.00	11.30 ± 4.70	9.04 ± 3.47	18.40 ± 6.82	<0.001

### Clinical information (anatomical-relevant classification)

Among the 129 cases, 76 cases were identified as common type, 15 cases as pharynx-invasion type, 18 cases as skull base-invasion type and 20 cases as mixed type, based on our anatomical classification. General clinical information, such as age, sex, site, and preclinical symptoms, did not differ among the four groups. Patients with the common type of tumor usually presented asymptomatic mass in the neck. Conversely, patients with the pharynx-invasion, skull base-invasion, and mixed types of tumors were more likely to present with throat discomfort or posterior cranial nerve dysfunction (Table 3).

**Table 3:** Clinical characters of anatomical-relevant classification

	Common type (*n* = 76)	Pharynx-invasion type (*n* = 15)	Skull base-invasion type (*n* = 18)	Mixed type (*n* = 20)	*P*-value
Age, years	46.00 ± 12.41	45.47 ± 12.77	41.22 ± 12.92	38.85 ± 11.95	0.097
Female/Male	43/33	6/9	10/8	11/9	0.703
Site (left/right)	40/36	8/7	10/8	12/8	0.948
Initial surgery	68 (89.47)	15 (100.00)	15 (83.33)	19 (95.00)	
Non-initial surgery	8 (10.53)	0	3 (16.67)	1 (5.00)	
Asymptomatic mass	37 (48.68)	6 (40.00)	7 (38.89)	8 (40.00)	0.791
Throat pain or discomfort	17 (22.37)	8 (53.33)	7 (38.89)	6 (30.00)	0.081
IX-XI cranial nerves dysfunction (Hoarseness, dysphagia, cough when drinking)	26 (34.21)	3 (20.00)	8 (44.44)	10 (50.00)	0.262
Tongue deviation	4 (5.26)	0	2 (11.11)	1 (10.00)	0.571

### Surgical outcomes (anatomical-relevant classification)

Representative pre-, intra-, and postoperative images of the different subgroups are shown in Fig. 4. Postoperative data are shown in Table 4. The majority of tumors were completely removed, while five cases classified as common type, four cases of skull base-invasion type and seven cases of mixed-type tumors received subtotal resection. The total resection rate was significantly lower for mixed-type tumors. Nine cases of common-type tumor received ECA ligation, the other ECA ligations occurred in patients with skull base-invasion and mixed-type tumors. ICA ligation was required in five cases with skull base-invasion and mixed-type tumors. ICA reconstruction with artificial vessels occurred in one case with skull base-invasion and in two cases with mixed-type tumors. ICA stent placement was also performed in one patient with a mixed-type tumor. The average bleeding and operation times in patients with skull base-invasion and mixed-type tumors were also significantly higher than those in the other groups. Two patients with skull base-invasion type and five with mixed-type tumors received ICU care because of ICA ligation or ICA laceration. The remaining patients were admitted to the ICU for other reasons as previously described. Postoperative stroke occurred in three patients. The incidence of cranial nerve impairment and subsequent lung infections in the four groups are compared in Table 4.

**Table 4:** Surgical outcomes based on anatomical-relevant classification

	Common type (*n* = 76)	Pharynx-invasion type (*n* = 15)	Skull base-invasion type (*n* = 18)	Mixed type (*n* = 20)	*P*-value
Total resection	71 (93.42)	14 (93.33)	15 (83.33)	13 (65.00)	0.006
Subtotal resection	5 (6.58)	0	4 (22.22)	7 (35.00)	0.006
ECA ligation	9 (11.84)	0	4 (22.22)	6 (30.00)	0.055
ICA ligation	0	0	1 (5.56)	4 (20.00)	<0.001
ICA Vascular patch	2 (2.63)	0	1 (5.56)	3 (15.00)	0.098
ICA reconstruction (Artificial vessel)	0	0	1 (5.56)	2 (10.00)	0.042
ICA reconstruction (Interventional stent)	0	0	0	1 (5.00)	0.139
Bleeding, ml	37.99 ± 43.21	98.33 ± 104.09	216.11 ± 128.30	468.00 ± 389.59	<0.001
Operation time, min	158.53 ± 56.23	218.87 ± 113.70	272.56 ± 132.11	436.30 ± 149.17	<0.001
ICU care	2 (2.63)	2 (13.33)	2 (11.11)	5 (25.00)	0.012
Stroke	0	0	1 (5.56)	2 (10.00)	0.042
IX-XI cranial nerves dysfunction (Hoarseness, dysphagia, cough when drinking)	10 (13.16)	6 (40.00)	4 (22.22)	4 (20.00)	0.102
Tongue deviation	3 (3.95)	1 (6.67)	2 (11.11)	2 (10.00)	0.591
Lung infection	1 (1.32)	2 (13.33)	3 (16.67)	4 (20.00)	0.010
Length of hospitalization, day	10.39 ± 3.86	11.33 ± 4.53	13.22 ± 6.26	15.60 ± 6.75	<0.001

## Discussion

CBTs are rare lesions in the PPS that exhibit distinct morphologies and growth characteristics. Because of their intimate relationship with the arteries and adjacent anatomical structures, CBTs are treated by ENT surgeons, vascular surgeons, and neurosurgeons according to their preferences [14, 15]. Unlike other solid tumors, resection of CBTs requires simultaneous consideration from two perspectives. The first is to remove the tumor from the PPS structures, such as the lateral wall of the pharyngeal cavity, jugular vein, hypoglossal nerve, vagus nerve, or glossopharyngeal nerve. The other is to safely dissociate the tumor from the carotid arteries. Shamblin group I–II CBTs are easily resected because of their small size and less-invasive features. However, the excision of III-CBTs is challenging because of their diverse vascular patterns and apparent aggressiveness. The classic Shamblin system fails to provide valuable guidance for the various forms of III-CBT, and a study has introduced a novel classification based on the arterial encasement and vertical extension of tumors [11]. However, that study ignored transverse pharyngeal invasion of the tumor and different vascular morphologies inside tumor. From a neurosurgeon’s perspective, we propose an updated classification system for III-CBTs to predict surgical outcomes and assist in developing appropriate strategies.

### Understandings based on arterial-relevant classification

III-CBTs arise from the bifurcation of the carotid arteries, with subsequent expansive development. The ECA and ICA are traditionally recognized as being compressed on either side of the tumor mass under the force of tumor growth. However, we found that some tumors exhibited eccentric growth tendencies that squeezed the ECA and ICA together on the same side. We classified these tumors as medial/lateral types when the ECA and ICA were located on the same side as the tumor body. The peculiar growth features unexpectedly provide great convenience during the operation, and surgeons do not have to search for the ECA and ICA within the tumor separately. The locations of both arteries can be easily confirmed based on their close relationships.

**Fig. 5:**
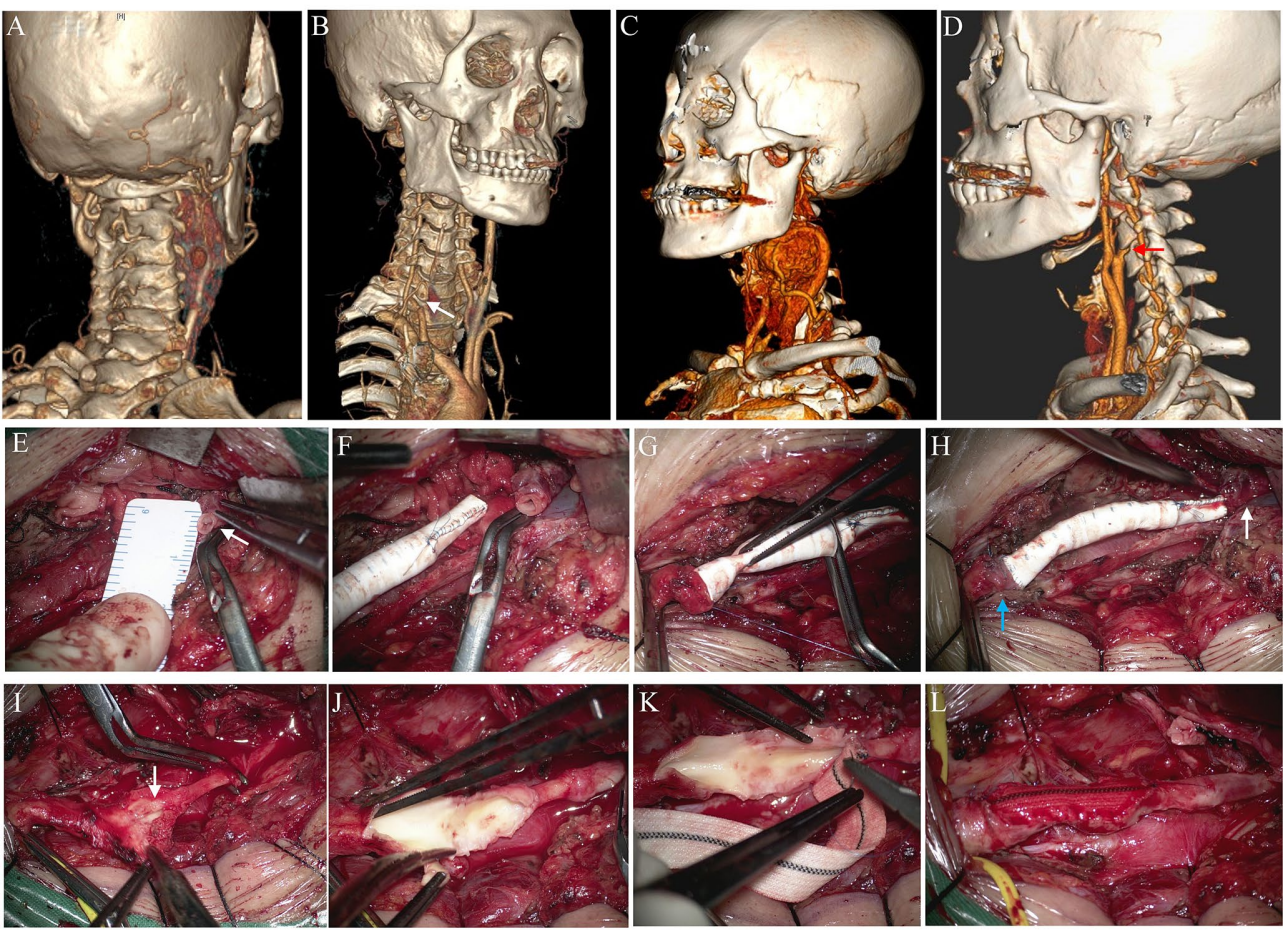
Different strategies to manage carotid arteries during operation. **A, B**. One case with both ligation of ICA and ECA. Preoperative CTA showed a large CBT with encased carotid arteries. Tumor was removed together with ICA and ECA. White arrow indicates the CCA stump. **C, D**. Another case with ligation of ECA and reservation of ICA. White arrow indicates the ECA stump. **E-H**. ICA reconstruction with artificial vessel. The distal end of ICA (white arrow) larger than 3 mm was connected with proximal end of CCA (blue arrow) by artificial vessel. **I-L**. ICA repair with vascular patch. A longitudinal cleft (white arrow) near the bifurcation part emerged during the removal of tumor. The ECA had already been ligated. **J, K**. The cleft was trimmed and enlarged for subsequent application of vascular patch. **L**. The ICA was successfully repaired

Extremely rare tumors with completely enveloped arteries cause significant difficulties and risks during surgery. If the ECA/ICA is located completely within the tumor, then the tumor is difficult or impossible to peel off. Forced dissection of the vessels often results in severe or unmanageable arterial bleeding. Therefore, comprehensive strategies should be developed for this type of tumor. For older patients, palliative surgery or subtotal resection is acceptable in order to protect the critical carotid arteries. For younger patients or when total resection is required, sufficient evaluation of the tumor site, volume, and distal end of the ICA is essential before surgery. If the tumor is relatively small, the upper margin is lower than C2, and the distal end of the ICA before entering the petrosal bone is sufficient for anastomosis, it is feasible to remove the tumor together with the enveloped ICA/ECA and connect the distal end of the ICA to the proximal end of the CCA using an artificial vessel. A distal ICA diameter of > 3 mm is considered suitable for vascular anastomosis. However, this also poses a high risk of failure or postoperative stroke because of thrombogenesis inside the artificial vessel. Antiplatelet treatment should be considered before surgery to avoid thrombosis if the arterial reconstruction strategy is preferred; however, antiplatelet therapies also increase the risk of bleeding during surgery. DSA and the BOT examination of the Willis circle function are essential for the enveloped type of tumor before surgery. It is possible to integrally incise the tumor and ICA together and directly ligate the carotid arteries if the circle of Willis functions well. The regurgitant blood flow and stump pressure of distal end of ICA act as ideal indicators of the circle of Willis function during surgery (Fig. 5).

Tumors around the bifurcation (initiation of the ICA/ECA) are regarded as the most adhesive in our clinical practice; therefore, we always dissect this part of the tumor last. Resection sometimes leads to a fine cleft or partial avulsion of the carotid arteries. Simple sutures are effective for treating small bleeding sites. The practicality of vascular patches has been proven in diverse carotid surgeries [16]. If the avulsed segment is relatively large, and a simple suture may lead to arterial stenosis, a vascular patch can be introduced for successful repair (Fig. 5). Experience and micro-suturing skills are necessary to treat complicated CBTs. Preoperative endovascular embolization was not essential in our cohort and the numerous feeding arteries were effectively controlled using bipolar cautery under a microscope. Therefore, it is reasonable to assume that neurosurgeons have a comparative advantage because of their micromanipulation performance.

### Understandings based on anatomical-relevant classification

The PPS is located between the internal pterygoid muscle, deep parotid gland, and lateral pharyngeal wall and is shaped like an inverted pyramid with its base at the skull base. The PPS is divided into pre-styloid and retro-styloid compartments by the styloid process. The pre-styloid space is small with ascending pharyngeal vessels crossing it. The retro-styloid space is large and contains the carotid artery, jugular vein, cranial nerves IX through XII, and the cervical sympathetic trunk. The space comes into direct contact with the skull base posterior to the styloid process and is continuous with the carotid sheath and the lateral wall of the pharynx. The adjacent relationships explain why masses in the carotid sheath frequently invade the skull base and pharyngeal wall [17, 18]. CBTs are the most common lesions of the PPS and have distinct growth patterns and imaging characteristics [19]. Based on the association between the tumor and anatomical structures in the PPS, we classified the III-CBTs into common, pharynx-invasion, skull base-invasion, and mixed types. The common and pharynx-invasion types share the same operative difficulties, and have relatively higher total resection rates. However, tumors of the pharynx-invasion type encroach on the pharyngeal wall, leading to additional complexities in perioperative management. If the pharyngeal cavity is severely compressed by the tumor, preoperative laryngoscopy is necessary for endotracheal intubation. Monitoring for postoperative dysphagia is also required, because of the disturbance/edema of the pharyngeal wall and superior/recurrent laryngeal nerve. Gastric catheterization can effectively reduce the risk of respiratory aspiration and serious lung infections.

The skull base-invasion type of tumor is difficult to remove because of the blockage of bony structures and neurovascular complexes. Piecemeal resection is recommended instead of en bloc resection for the treatment of this type of tumor. A large proportion of the tumor located away from the skull base should first be resected to release the surgical space. The residual parts of the tumor near the skull base can then be carefully removed piece-by-piece under high magnification. Piecemeal resection usually leads to massive bleeding; therefore, extreme caution is required when treating tumors around the skull base. A limited operative field and vision greatly increase the risk of damage to the vessels and cranial nerves. Intraoperative fluorescein angiography aids in distinguishing between the tumor tissue and carotid arteries. The fascia near the styloid process should be well-protected to avoid injury to the facial nerve.

Mixed-type tumors with extensive invasion of the pharynx and skull base are the most challenging lesions for surgeons. This type of tumor has the lowest rate of total resection and the highest rate of complications. Piecemeal or palliative resection is acceptable for a mixed-type tumor, to avoid lethal bleeding or neurological dysfunction. Preoperative DSA and the BOT are required to determine the potential for vascular ligation and reconstruction. Long-term endotracheal intubation, gastric catheterization and ICU care may be necessary in patients with mixed-type tumors as consequences of severe laryngeal edema, posterior cranial nerves dysfunction and stroke.

Regular curved incisions along the anterior border of the SCM are sufficient to expose all types of III-CBTs, in our clinical experience, even for skull base-invasion and mixed-type tumors. The post-auricular end of the incision can be greater than the mandibular angle to avoid the parotid gland and fully expose the skull base. Surgical draw hooks were introduced to maximize the operative field as much as possible. Microscopy is the principal method of exposing portions of tumors that are limited by the skull base. Surgeons can observe the skull base directly by obliquely switching the position and angle of the microscope. High magnification favors the identification of residual tumor and vessel boundaries within the scope of the skull base. Mandibular osteotomy [20] is unnecessary for tumors that infiltrate the skull base during surgery.

### Pseudocapsule

**Fig. 6:**
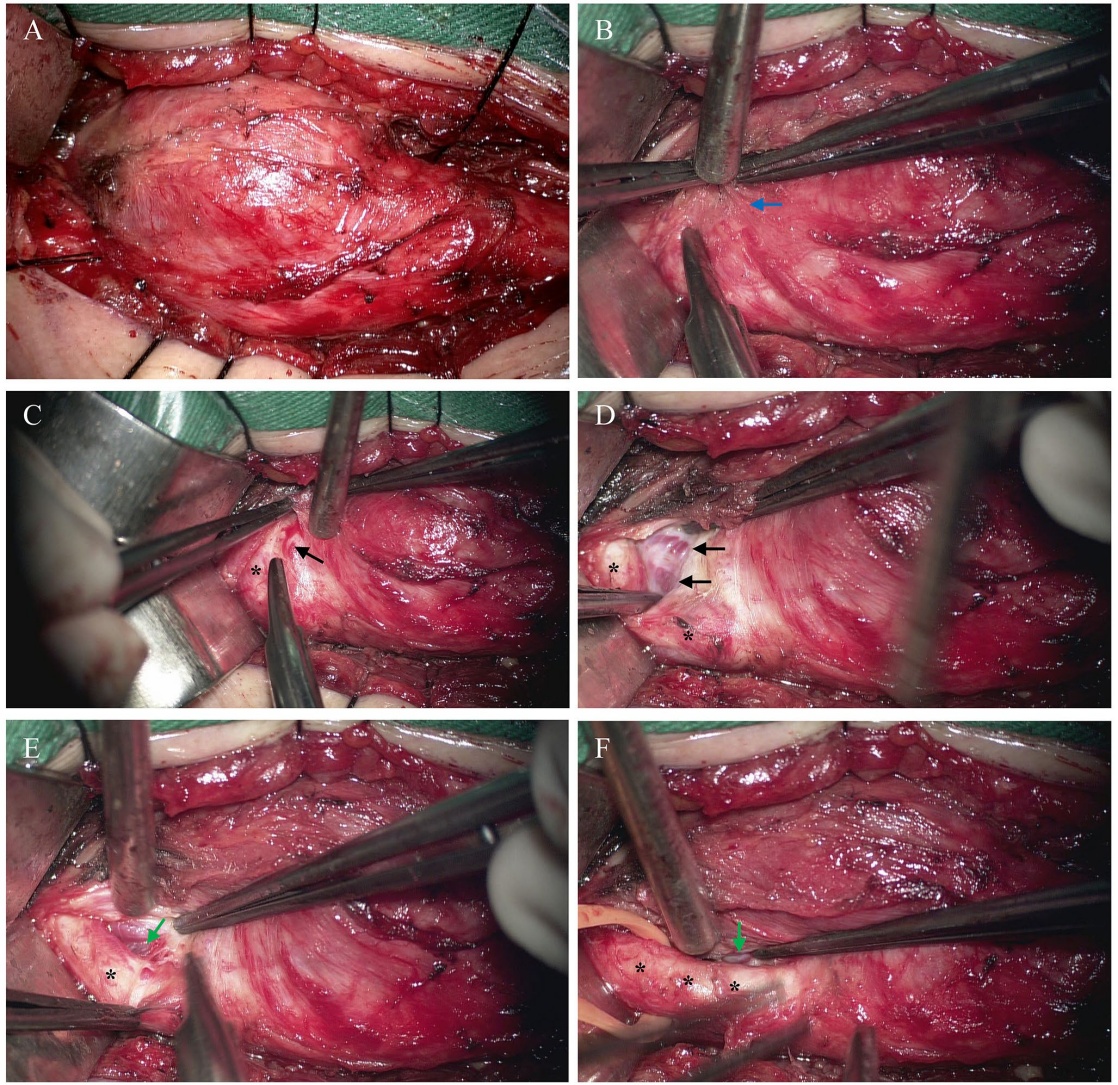
The pseudocapsule and “potential space” between tumor and carotid arteries. (**A**) The integral view of CBT in PPS. It’s hard to distinguish carotid arteries and tumor due to the coverage of pseudocapsule. (**B**) The pseudocapsule (blue arrow) is a thin layer of loose fibrous tissue which is dissected to expose the hidden carotid arteries and tumor bulk. **C, D**. The tiny and tortuous feeding arteries (black arrow) are exposed after removal of pseudocapsule. **E, F**. The “potential space” (green arrow) appears after electrocoagulation of feeding arteries. Tumor and ICA can be separated clearly along the space. *: ICA.

The concept of a pseudocapsule of III-CBTs has not been proposed before (Fig. 6). The pseudocapsule is the outer fibrous layer that partially covers the solid part of the tumor and carotid arteries in III-CBTs. After exposure of the CBT in the PPS, it is difficult to determine the exact position of the carotid arteries because of the existence of a pseudocapsule, even though the arteries are located at the border of the tumor. Pseudocapsules are more likely to appear in tumors with a relatively soft texture. The pseudocapsule layer can be dissected to expose the hidden carotid arteries and tumor tissues, especially in classical CBTs. The potential space between the pseudocapsule layer, tumor, and carotid arteries contains tortuous and dilated feeding arteries, which explains the extremely sufficient blood supply to the tumor that can be utilized for dissociation. Tortuous arteries are controlled by bipolar cautery to decrease tumor blood perfusion. Subcapsular bipolar hemostasis combined with blunt and sharp dissections are the most effective procedure for CBT removal. Understanding and recognizing the pseudocapsule structure is beneficial for accurate dissection and reduced bleeding. However, a small portion of tumors are exceptionally difficult to dissect because of their hard texture. The pseudocapsule and potential space are always disappeared in this type of tumor. The solid tumor tissues broadly invade and squeeze the potential space, which further increases the difficulty of surgery. In these cases, intraoperative fluorescein angiography under a microscope allows precise identification of the carotid arteries. Progressive fluorescence imaging during surgery is essential in complicated cases, especially for the enveloped and mixed types of tumors.

### Re-operation cases

Some patients have received secondary treatment at our center, which brings more difficulties and risks for surgeons because of scar formation and disorganized anatomical structures. A detailed MRI scan is required to determine the exact location of the CCA and the internal jugular vein before surgery. Anatomical adhesions are hidden in the space between the deeper layer of the SCM and the superficial layer of the tumor, which should be carefully dissociated to avoid damage to the vessels and cranial nerves. The pseudocapsule structure also disappears in the adhesive region. However, structures at deeper sites of the tumor were not disturbed in most reoperation cases, with the pseudocapsules remaining intact. The pseudocapsule provides a clear anatomical interface for carotid artery identification and tumor resection.

## Conclusion

We have presented a summary of a III-CBT series from our single center. We have proposed a novel PKU classification of III-CBTs from the perspective of arterial morphology and anatomical differences. These novel classifications enable surgeons to comprehensively understand the potential risk of tumors and plan the appropriate management for each case. The existence of a pseudocapsule should be noted and utilized during surgery.

## Data Availability

No datasets were generated or analysed during the current study.
